# Logistic regression over encrypted data from fully homomorphic encryption

**DOI:** 10.1186/s12920-018-0397-z

**Published:** 2018-10-11

**Authors:** Hao Chen, Ran Gilad-Bachrach, Kyoohyung Han, Zhicong Huang, Amir Jalali, Kim Laine, Kristin Lauter

**Affiliations:** 1 Microsoft Research, Redmond, WA USA; 2 Seoul National University, Seoul, Korea; 3 École Polytechnique Fédérale de Lausanne, Lausanne, Switzerland; 4 Florida Atlantic University, Boca Raton, USA

**Keywords:** Cryptography, Homomorphic encryption, Logistic regression

## Abstract

**Background:**

One of the tasks in the 2017 iDASH secure genome analysis competition was to enable training of logistic regression models over encrypted genomic data. More precisely, given a list of approximately 1500 patient records, each with 18 binary features containing information on specific mutations, the idea was for the data holder to encrypt the records using homomorphic encryption, and send them to an untrusted cloud for storage. The cloud could then homomorphically apply a training algorithm on the encrypted data to obtain an encrypted logistic regression model, which can be sent to the data holder for decryption. In this way, the data holder could successfully outsource the training process without revealing either her sensitive data, or the trained model, to the cloud.

**Methods:**

Our solution to this problem has several novelties: we use a multi-bit plaintext space in fully homomorphic encryption together with fixed point number encoding; we combine bootstrapping in fully homomorphic encryption with a scaling operation in fixed point arithmetic; we use a minimax polynomial approximation to the sigmoid function and the 1-bit gradient descent method to reduce the plaintext growth in the training process.

**Results:**

Our algorithm for training over encrypted data takes 0.4–3.2 hours per iteration of gradient descent.

**Conclusions:**

We demonstrate the feasibility but high computational cost of training over encrypted data. On the other hand, our method can guarantee the highest level of data privacy in critical applications.

## Background

Since 2014, iDASH (integrating Data for Analysis, Anonymization, and Sharing) has hosted yearly international contests around the theme of genomic and biomedical privacy. Teams from around the world participate to test the limits of secure computation on genomic and biomedical tasks, and benchmark solutions on real data sets. Such contests serve to bring together experts in security, cryptography, and bioinformatics to quickly make progress on interdisciplinary challenges. The task for outsourced storage and computation this year was to implement a method for private outsourced training of a logistic regression model.

### Motivation

Machine Learning (ML) over encrypted data has important applications for cloud security and privacy. It allows sensitive data, such as genomic and health data, to be stored in the cloud in encrypted form without losing the utility of the data. For the third task in the 2017 iDASH Secure Genome Analysis Competition, participants were challenged to train a machine learning model on encrypted genomic data that will predict disease based on a patient’s genome. In a non-interactive (with outsourced storage) setting, training ML models on encrypted data had up until now only been done for very simple models, such as Linear Means Classifiers and Fisher’s Linear Discriminant Analysis [1]. Interactive settings, where multiple parties hold shares of the data and communicate throughout the training process, have been developed for several more complicated models, but they require high communication costs and a non-colluding assumption between several clouds [2].

The 2017 iDASH competition task was to train a logistic regression model, and although in theory it can be done using Fully Homomorphic Encryption (FHE) [3, 4], until now the feasibility and efficiency of this approach had not been studied.

### Summary of results

In this work, we show that training a logistic regression model over binary data is possible using FHE. In particular, we use gradient descent and stochastic gradient descent algorithms with mini-batches, and demonstrate that it takes several minutes to one hour to run each gradient descent step. Our solution can run for an arbitrary number of steps, as opposed to the now commonly used practical homomorphic encryption (PHE) approach [5], where the size of the computation is determined beforehand, and parameters chosen once and for all to support a computation of that size. This is possible using Craig Gentry’s bootstrapping operation [4], which we have implemented for the first time for the Fan-Vercauteren scheme [6] using the publicly available homomorphic encryption library SEAL (http://sealcrypto.org; accessed on 9 April, 2018).

More precisely, in fully homomorphic encryption each ciphertext contains a component called the noise, which grows in all homomorphic operations, and eventually reaches a maximum value. Once this maximum is reached, the ciphertext cannot be decrypted correctly anymore. Bootstrapping is the process of “refreshing” FHE ciphertexts to reduce the noise levels during deep computations to ensure correct decryption at the end of the computation.

Another challenge in the approach we take is the plaintext data type supported by the homomorphic encryption scheme. Namely, it is only possible to encrypt fairly small integers with SEAL, and indeed with many homomorphic encryption schemes. In machine learning the model weights are typically rational numbers, which need to be scaled to integers. Unfortunately, this quickly causes an overflow to occur in our rather small integer data type, unless the integers can be scaled down. We describe a modified bootstrapping operation which merges bootstrapping and such a scaling into one step, significantly reducing the complexity of our algorithm.

Besides noise growth and message expansion, another challenge in implementing Logistic Regression with FHE is applying the sigmoid function. We present two methods to approximate this function with a polynomial, and compare them both in terms of the accuracy of the trained model and in terms of computation time.

### Related work

At the time of writing this, very little directly comparable prior work exists. The closest to our approach is [7], where the authors achieve remarkably good performance in training small logistic regression models; in their solution it is necessary that the number of features is very small (logarithmic in the number of training records).

A slightly different approach is taken in [8], where the authors use the homomorphic encryption library HEAAN, that natively supports scaling down of plaintext numbers [9, 10]. The authors report good performance numbers, but unlike us and [7] they only allow a very small number of iterations. Extending to more iterations will be computationally very costly, and require bootstrapping.

## Methods

### Fan-Vercauteren scheme

Fully Homomorphic Encryption (FHE) refers to a type of encryption scheme, envisioned already a few decades ago [3], that allows arbitrary computations to be performed directly on encrypted data. A blueprint for a solution was first proposed by Gentry [4] in 2009, and since then numerous schemes have been proposed. In this work we use the Fan-Vercauteren scheme (FV) [6], and its implementation in the SEAL library [11].

#### Parameters and notation.

We start by defining the parameters of the FV scheme. Let *q*≫*t* be positive integers and *n* a power of 2; often *t* is a prime such that 2*n*∣(*t*−1). Denote *Δ*=⌊*q*/*t*⌋. We define $R = {\mathbb {Z}}/(x^{n}+1)$, $R_{q} = {\mathbb {Z}}_{q}[x]/(x^{n}+1)$, and $R_{t} = {\mathbb {Z}}_{t}[x]/(x^{n}+1)$. Here, ${\mathbb {Z}}$ is the set of polynomials with integer coefficient and ${\mathbb {Z}}_{q}[x]$ is the set of polynomials with integer coefficient in range [0,*q*−1). Therefore, *R*_*q*_ is the set of polynomials of degree at most *n*−1, with coefficients integers modulo *q*. Multiplications of polynomials in *R*_*q*_ is similar to usual polynomial multiplication, except that *x*^*n*^ should in every step be replaced by − 1. In the FV scheme plaintext elements are polynomials in *R*_*t*_, and ciphertext elements are pairs of polynomials in *R*_*q*_×*R*_*q*_. Let *χ* denote a narrow (centered) discrete Gaussian error distribution. In practice, most implementations of homomorphic encryption use *σ*[*χ*]≈3.2. Finally, let *U*_*k*_ denote the uniform distribution on ${\mathbb {Z}} \cap [-k/2, k/2)$.

#### Key generation

The first step in using the FV scheme is generating a public-secret key pair (pk,sk). To do this, sample $s \leftarrow U_{3}^{n}$, $a \leftarrow U_{q}^{n}$, and *e*←*χ*^*n*^; here *s*, *a*, and *e* are all considered as elements of *R*_*q*_, where the *n* coefficients are sampled independently from the given distributions. To form the keys, we let

$$\texttt{pk} = ({[-(as + e)]}_{q}, a) \in R_{q}^{2} \,, $$

where [·]_*q*_ denotes the (coefficient-wise) reduction modulo *q*. In reality there are other types of keys involved, in particular so-called evaluation keys and Galois keys, but for the sake of simplicity we will omit discussing them here, and refer the reader to [6, 11].

#### Encryption.

Let *m*∈*R*_*t*_ be a plaintext message. To encrypt *m* with the public key $\texttt {pk} = (p_{0}, p_{1})\in R_{q}^{2}$, sample $u \leftarrow U_{3}^{n}$, $a \leftarrow U_{q}^{n}$, and *e*_1_,*e*_2_←*χ*^*n*^. Consider *u*, *a*, and *e*_*i*_ as elements of *R*_*q*_ as in key generation, and create the ciphertext

$$\texttt{ct} = ([\Delta m + p_{0} u + e_{1}]_{q}, [p_{1} u + e_{2}]_{q}) \in R_{q}^{2} \,. $$

#### Decryption.

To decrypt a ciphertext ct=(*c*_0_,*c*_1_) given a secret key sk=*s*, write

$$\frac{t}{q}(c_{0} + c_{1} s) = \widehat{m} + v + bt \,, $$

where *c*_0_+*c*_1_*s* is computed as an integer coefficient polynomial, and scaled by the rational number *t*/*q*, *b* is an integer coefficient polynomial, $\widehat {m}$ the underlying message, and *v* the leftover fractional part.

It is easy to see that when *q* is sufficiently larger than *t*, then $\widehat {m} = m$, and ∥*v*∥_*∞*_≪1/2. This means that the original message can be recovered by computing

$$m = \left\lfloor \frac{t}{q}(c_{0} + c_{1} s)\right\rceil_{t} \,, $$

where ⌊·⌉ denotes rounding to the nearest integer. For details, see [6, 11].

#### Homomorphic computations

A final fundamental piece in the puzzle is how to enable additions and multiplications of two ciphertexts. For addition, this is easy; we define an operation ⊕ between two ciphertexts ct_1_=(*c*_0_,*c*_1_) and ct_2_=(*d*_0_,*d*_1_) as follows:

$$\texttt{ct}_{1} \oplus \texttt{ct}_{2} = ([c_{0} + d_{0}]_{q}, [c_{1} + d_{1}]_{q}) \in R_{q}^{2} \,. $$

We denote this homomorphic sum by $\texttt {ct}_{\text {sum}} = (c^{\text {sum}}_{0}, c^{\text {sum}}_{1})$, and note that if

$${} \frac{t}{q}(c_{0} + c_{1} s) = m_{1} + v_{1} + b_{1} t \,,\quad \frac{t}{q}(d_{0} + d_{1} s) = m_{2} + v_{2} + b_{2} t \,, $$

then

$$\frac{t}{q}(c^{\text{sum}}_{0} + c^{\text{sum}}_{1} s) = [m_{1} + m_{2}]_{t} + v_{1} + v_{2} + b_{\text{sum}} t \,, $$

as long as ∥*v*_1_+*v*_2_∥_*∞*_<1/2. Thus, ⊕ passes through the encryption to the underlying plaintexts, and results in an encryption of the sum [*m*_1_+*m*_2_]_*t*_ as long as ∥*v*_1_+*v*_2_∥_*∞*_<1/2.

It is similarly possible to define an operation ⊗ between two ciphertexts, that results in a ciphertext decrypting to [*m*_1_*m*_2_]_*t*_, as long as ∥*v*_1_∥_*∞*_ and ∥*v*_2_∥_*∞*_ are small enough. Since ⊗ is much more difficult to describe than ⊕, we refer the reader to [6, 11] for details.

#### Noise

In the decryption formulas presented above the rational coefficient polynomials *v* are assumed to have small enough infinity-norm, namely less than 1/2. This is clearly necessary, as otherwise the ciphertext will result in the incorrect plaintext being recovered. Given a ciphertext ct=(*c*_0_,*c*_1_) encrypting a plaintext *m*, let $v \in \mathbb {Q}[x]/(x^{n} + 1)$ such that

$$\frac{t}{q}(c_{0} + c_{1} s) = m + v + bt \,. $$

The polynomial *v* is called the noise polynomial, ∥*v*∥_*∞*_ is called the noise, and the ciphertext decrypts correctly as long as the noise is less than 1/2 [11].

When operations such as addition and multiplication are applied to encrypted data, the noise in the result may be larger than the noise in the inputs; this is referred to as noise growth. This noise growth is very small in homomorphic additions, but substantially larger in homomorphic multiplications. Thus, given a specific set of encryption parameters (*n*,*q*,*t*,*χ*), one can only evaluate computations of a bounded size (in practice, of bounded multiplicative depth), until the noise grows too large making the ciphertext impossible to decrypt even with the correct secret key.

To mitigate the problem of high noise growth rates Craig Gentry [4] described a clever approach which is commonly known as bootstrapping. In this process, an encrypted version of the secret key is used to decrypt the message using homomorphic operations. Therefore, the result of this process is similar to a freshly encrypted message and hence it has only a small amount of noise. This bootstrapping process is considered to be a very costly operation in most schemes [10, 12, 13], but not in all [14, 15].

### Batching

The FV scheme (and many other homomorphic encryption schemes) inherently support SIMD operations. This capability is commonly called “batching” in literature, and is explained in detail e.g. in [11] in the context of the SEAL library that we use.

The idea is that by choosing the plaintext modulus *t* appropriately, the plaintext space *R*_*t*_ is isomorphic as a ring to the *k*-fold product $\mathbb {F}_{t^{n/k}} \times \ldots \times \mathbb {F}_{t^{n/k}}$, for some *k*∣*n*. In other words, operations in *R*_*t*_ translate automatically into *k* concurrent operations in the extension field $\mathbb {F}_{t^{n/k}}$, for example allowing us to perform *k*-fold SIMD operations on integers up to *t* by using only the subfield ${\mathbb {Z}}_{t} \subset \mathbb {F}_{t^{n/k}}$.

Using batching efficiently can be non-trivial, and typically requires one to carefully design the computation to maximize the benefit.

### Logistic regression

Logistic Regression is a common tool used in machine learning to build a model that can discriminate between samples from two or more classes. It arises from the need to model the posterior probabilities of *K* classes via linear functions of input $x \in {\mathbb {R}}^{D}$. In this work we consider two-class classification, so *K*=2. To simplify the notation, we assume the input vector *x* always has 1 as the first element, which accounts for the bias term in the linear function. Then the logistic regression model has the form

$$ \log \left[ {\frac{\text{Pr}(Y = 0 \mid X = x)}{\text{Pr}(Y = 1 \mid X = x)}} \right] = w^{T} x\,, $$

where *Y* denotes the class, and $w \in {\mathbb {R}}^{D}$ is the weight vector that we need to learn in model training. The above model is specified in terms of log-odds ratio, reflecting the constraint that the probabilities sum to one. An alternative and more common form is to represent it as the following posterior probability for class 0:

$$ \text{Pr}(Y = 0 \mid X = x) = \frac{1}{ 1 + e^{-w^{T} x}} = \sigma(w^{T} x) \,, $$

where *σ*(*t*)=1/(1+*e*^−*t*^) is known as the sigmoid function. Next we present two algorithms for learning *w*.

### Training algorithms

Our goal is to evaluate a training algorithm for a logistic regression model on homomorphically encrypted data. In this section we present the two training algorithms that we evaluated for this purpose.

#### Gradient descent

The standard method for training logistic regression is gradient descent. To fix notation, let *D* be the number of (binary) features, and *N* the number of training records of the form (*X*,*y*), where $X \in {\mathbb {R}}^{N \times D}$, $y \in {\mathbb {R}}^{N}$. In this case the weight vector *w* is in ${\mathbb {R}}^{D}$.

Gradient descent proceeds in iterations, where in each iteration the weight vector *w* is updated as

$$w \leftarrow w - \alpha (\sigma(Xw) - y) X^{T}\,, $$

where *σ* is the sigmoid function, and *α*>0 a learning rate parameter. We formalize the gradient descent algorithm below.


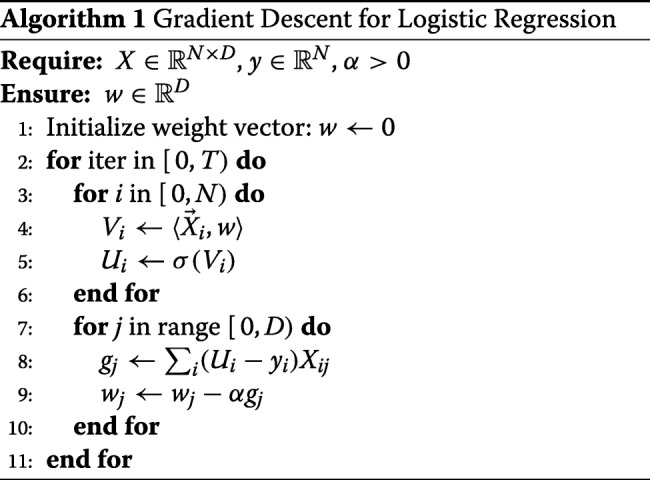


#### 1-bit gradient descent

A direct application of Algorithm 1 suffers from the problem of quickly growing plaintext size—a problem which was briefly mentioned in “Summary of results”. Namely, the parameter *t* in the homomorphic encryption scheme is typically quite small, causing integer plaintext data to quickly become reduced modulo *t*. This is similar to the problem using a too small data type in normal programming, except that in this case it is difficult to switch to a larger one. For this reason, we need to be able to control the growth of our encrypted numbers either by scaling them down, and/or by designing our computation in a way that minimizes the increase in the size of the numbers.

For the first approach, we need a homomorphic floor function, which we discuss in “Fixed point arithmetic over plaintext data”. For the second approach, we note that multiplying by just a sign never increases the size of a number, so replacing one multiplicand by its sign allows the plaintext size to remain much smaller. Unfortunately, homomorphic sign extraction is very difficult, but turns out to be still faster than the homomorphic floor function. For this reason, we opt to use sign information instead of evaluating floor function to make our homomorphic training faster. By using the 1-Bit Gradient Descent (1-Bit GD) algorithm, which was invented to compress the gradient in order to reduce communication during training [16], our homomorphic training becomes much faster.

In the 1-Bit GD method, in each iteration we update each weight by a learning rate multiplied by the sign of the corresponding coordinate of the current gradient, plus a residue term. The unused part of the gradient is then added back into the residue. We also introduce a new parameter *β*, which reduces the magnitude of the accumulated residues in the past. Our modified 1-Bit GD is presented formally in Algorithm 2.


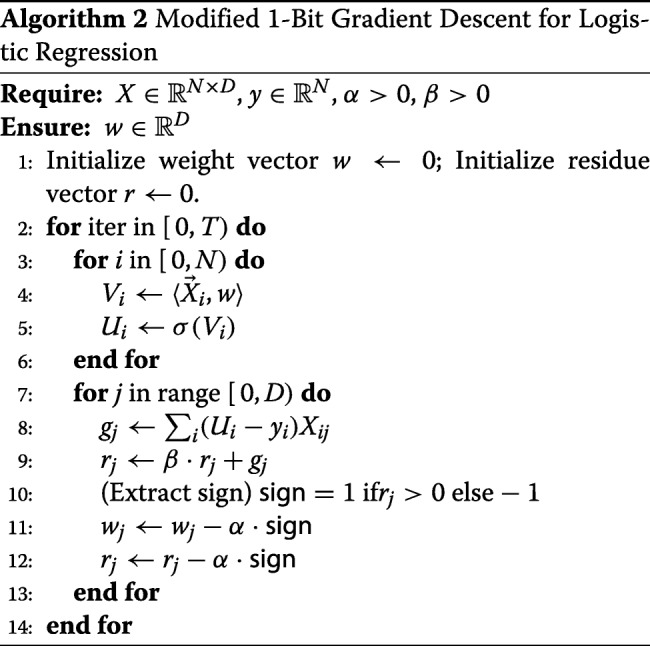


The 1-Bit GD approach can be done easily also in the stochastic setting, where either individual records or mini-batches are processed at a time. In this work, for the sake of simplicity, we will only focus on full gradient descent.

### Fixed point arithmetic

#### Fixed point arithmetic over plaintext data

Logistic Regression is naturally performed over floating point numbers. However, in the FV scheme there is no easy way to encrypt numbers of this type directly, so they need to be first scaled to integers of some fixed precision.

In fixed point number representation we choose an integer base *p* (in this work we will fix *p* to be an odd prime), the number of integral digits *l*, and the number of fractional digits *f*. Then a fixed point number is a rational number *x* of the form

$$ x = \sum\limits_{i = -f}^{l-1} x_{i} p^{i}\,, $$

with $x_{i} \in [-(p-1)/2,\ldots, (p-1)/2] \cap {\mathbb {Z}}$. That is, every fixed point number has *l* integral digits and *f* fractional digits in base *p*. We need *f* extra digits to hold an intermediate result from multiplication, hence we let *r*=*l*+2*f* and set the modulus to be *p*^*r*^ (see also [17]). To encode a number, we multiply by *p*^*f*^ and round to an integer, i.e. the representation of *x* is $\tilde {x} = p^{f} x$. See

To add/subtract two fixed numbers, we simply add/subtract their representations modulo *p*^*r*^. To multiply two fixed point numbers *x* and *y*, we compute

$$\tilde{z} = \left\lfloor \frac{\tilde{x} \tilde{y} \pmod{p^{r}}}{p^{f}} \right\rfloor \,. $$

Note that although standard fixed point arithmetic requires us to perform scaling after every multiplication, it is not strictly needed. For example, if we are going to compute $\sum _{i=1}^{n} x_{i} y_{i}$, then it is possible to not scale after each product, but only scale after the sum. This may not save a lot of work over plaintext, since scaling is fast; however, since scaling is expensive over encrypted data, this technique is useful in our setting.

#### Bootstrapping

Even for relatively small examples, Algorithm 1 and Algorithm 2 result in (multiplicatively) high-depth arithmetic circuits; the depth is equal to the number of iterations times the depth of a single iterative step. Recalling the noise growth problem discussed above in “Noise”, a straightforward implementation will have to use bootstrapping regularly to maintain the correctness of the final result. Since bootstrapping is a costly operation, we introduce below in “Combining bootstrapping with scaling” a modification to this step that does both the noise cleaning and also scaling, which is used to prevent plaintext size expansion.

We modified the bootstrapping algorithm from [13], where the crucial part of the bootstrapping procedure is a homomorphic digit removal process. Namely, suppose the plaintext modulus of our homomorphic encryption scheme is a prime power *t*=*p*^*r*^, and the plaintext is (for simplicity) just an integer $m \in {\mathbb {Z}}_{p^{r}}$. Then as an intermediate result in bootstrapping we have an encryption of *M*=*p*^*e*−*r*^*m*+*v*, where *e*>*r*, *p*^*e*^ is an intermediate plaintext modulus, and |*v*|<*p*^*r*^/2 is the noise to be removed. If we have a polynomial which removes the lowest *e*−*r* digits in an integer modulo *p*^*e*^, then applying it to *M* will give us *p*^*e*−*r*^*m*, which is a scalar multiple of the original message. In the FV scheme the scalar multiple can be easily removed when the plaintext modulus is divided by the scalar value. So the bootstrapping procedure finishes by removing the scalar value. Below in “Combining bootstrapping with scaling” we apply these ideas to achieve bootstrapping together with scaling down of encrypted numbers, resulting in encrypted fixed point arithmetic.

#### Combining bootstrapping with scaling

In order to perform the scaling functionality over encrypted data, we need to express the functionality as a polynomial. This is possible, however the polynomial will often have large degree, forcing us to perform bootstrapping to refresh the noise after each scaling over encrypted data. It turns out that these two steps can be combined for improved performance.

Suppose we have an encryption of a message *m* modulo *p*^*r*^, and we wish to obtain an encryption of ⌊*m*/*p*^*i*^⌋. First, we can apply a free division operation in FV (see e.g. [17]) to obtain an encryption of ⌊*m*/*p*^*i*^⌋+*p*^*r*−1^*α* with full noise, where *α* represents some “upper garbage”. Then we perform modulus-switching followed by a dot product with the bootstrapping key (see e.g. ([13], Section 4.1)) to obtain a low-noise encryption of *v*+*p*^*e*−*r*^⌊*m*/*p*^*i*^⌋+*p*^*e*−*i*^*α* (mod *p*^*e*^), with |*v*|≤*p*^*e*−*r*^/2. Then we follow the bootstrapping algorithm and homomorphically evaluate a polynomial of degree *ep*^*e*−*r*^ to remove the *v* term. Finally, we apply one extra step to remove the *α* term. This can be done in a similar fashion, by evaluating a digit removal polynomial of degree *rp*^*r*−*i*^. As a result, we obtain an encryption of ⌊*m*/*p*^*i*^⌋. We will use FHE.bscale(·,*i*) to denote the above bootstrapping plus scaling down by *i* digits in base *p*. For convenience of notation, we set the default value of *i* to be 1. The total degree of the procedure is *ep*^*e*−*r*^·*rp*^*r*−*i*^=*erp*^*e*−*i*^.

## Results

In this section we describe experiments with the techniques described in previous sections.

### Dataset description

We used two datasets to test the performance of our homomorphic machine learning algorithm.

#### iDASH 2017 competition dataset

The dataset provided by the iDASH competition organizers consists of 1579 training samples, where each sample contains a binary phenotype (cancer/no cancer), and 108 binary genotypes. In the evaluation of the solution, the organizers selected 18 genotypes to use as the features and therefore, in the experiments reported below only these 18 features were used.

#### MNIST dataset

The MNIST dataset [18] consists of hand written digits, stored as images, and it is commonly used as benchmark for machine learning systems. Each image in the original dataset is a 28×28 pixel map, where each pixel is represented in a 256 level gray-scale code. We first selected 1500 images containing handwritten digits ‘3’ and ‘8’ to obtain a binary classification problem. Then we compressed each image into 196 features with each feature an integer in the range [0,8), by dividing each pixel value by 32 and performing average pooling with window of size 2×2.

### Parameter selection

Selecting the right parameters can make a big difference in performance in terms of speed, space, and accuracy. Here we described the parameter tuning performed in the experiments.

#### FHE parameters

The FHE parameters need to be chosen carefully in order to achieve correctness, security, and performance. There are three crucial FHE parameters to be chosen: the ring dimension *n*, the ciphertext modulus *q* and the plaintext modulus *t*.

Smaller *n* and *q* imply better speed, while in order to support bootstrapping and scaling operations *n* and *q* need to be sufficiently large. In our experiments we chose *n*=2^15^ and *q*≈2^1020^, as these parameters are just large enough for bootstrapping and scaling, yet as small as possible for optimal performance. More precisely, we chose *q* as a product of 17 primes—each 60 bits in size—as required by SEAL. These parameters guarantee around 100 bits of security.

The value of *t* determines the precision of our computation: the larger *t* is, the more correct digits we will expect to see in the result. On the other hand, if *t* is too large, bootstrapping and scaling cannot be supported unless we also increase the value of *q*. We chose to use *t*=*p*^*r*^=127^3^ to balance between precision and performance. This configuration supports 64 slots per ciphertext (recall “Batching”, and see “Data batching method” below).

#### ML parameters

We use two training algorithms. The first one uses a linear approximation of the sigmoid function together with 1-bit GD (Algorithm 2), while the second algorithm uses a degree 3 approximation of the sigmoid function and normal gradient descent (Algorithm 1). Note that we chose to use a linear approximation of the sigmoid function in the 1-bit GD method, because there is no need to use higher degree approximation due to only the sign being considered. For the iDASH dataset we let the training algorithm perform 36 iterations over the training data, while for the MNIST dataset we perform 10 iterations. For the iDASH dataset, the learning parameters were set to *α*=0.1 and *β*=0.2 for Algorithm 2, and *α*=0.0002 for Algorithm 1. For the MNIST data set, we used *α*=0.01 and *β*=0.2 for Algorithm 2, and *α*=10^−5^ for Algorithm 1.

#### Approximating the sigmoid function

There are several methods to find an approximate polynomial for a given function. The best known method is probably Taylor polynomials, but it minimizes the error only in the vicinity of one point. For this reason, we instead use an approach similar to [19], and use a so-called minimax approximation.

Let *P*_*d*_ denote the set of polynomials of degree at most *d*, and for a continuous function *f*∈*C*[*a*,*b*] denote ∥*f*∥=max{|*f*(*x*)|:*x*∈[*a*,*b*]}.

##### **Definition 1**

*p*∈*P*_*d*_ is a *d*-th minimax approximation of *f*∈*C*[*a*,*b*] if

$$ \| f - p \| = \inf\{ \| f-q \| : q \in P_{d} \}. $$

For more details, we refer the reader to [20].

A minimax approximation algorithm (or uniform approximation) is a method to find the polynomial *p* in the above definition. The Remez algorithm [21] is an iterative minimax approximation algorithm, and yields the following results for the interval [−5,5] and degrees 1 and 3:

$${}\sigma_{1}(x) = 0.125x + 0.5\,, \quad \sigma_{3}(x) = -0.004x^{3} + 0.197x + 0.5 \,.$$

**Fig. 1:**
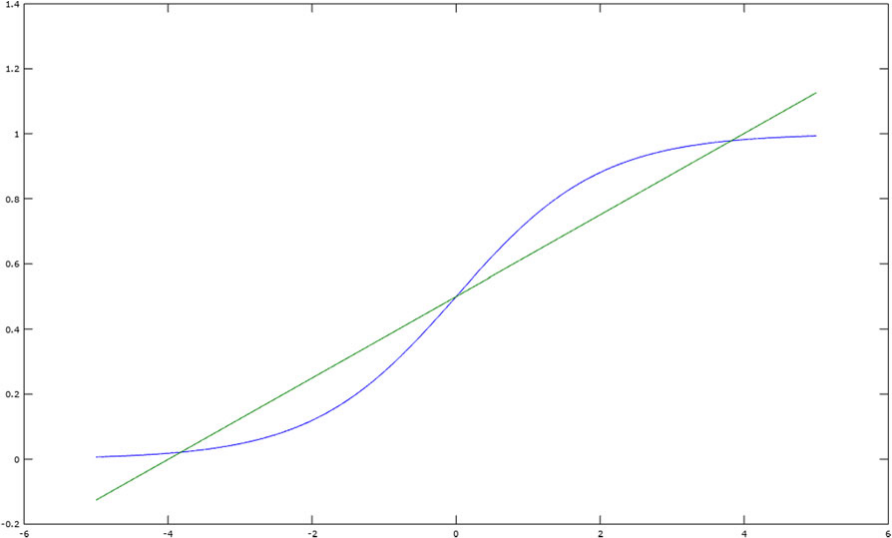
Linear minimax approximate for sigmoid: *f*(*x*)=0.5+0.125*x*

**Fig. 2:**
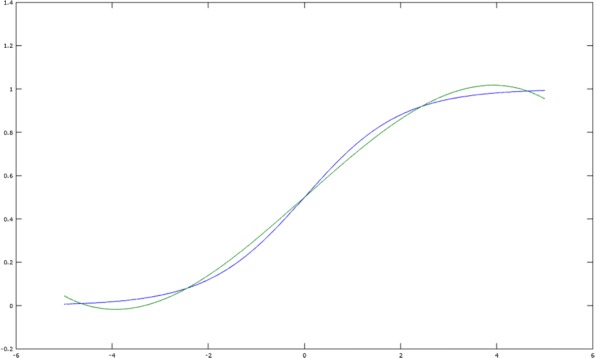
Degree 3 minimax approximate for sigmoid: *f*(*x*)=0.5+0.197*x*−0.004*x*^3^

These functions are illustrated in Fig. 1 and Fig. 2, respectively.

### Data batching method

In order to efficiently use the batching capabilities in SEAL (recall “Batching”), we encode the training dataset “vertically”, i.e. each ciphertext will store one single genotype/phenotype from *k* samples, where *k* is the number of slots in one plaintext. For example, the FHE parameters presented above in “Parameter selection” yield *k*=64 slots. On the other hand, we will need *D* plaintexts to represent the weights, where within each plaintext vector the weight is repeatedly encoded *k* times. As a result, the data matrix *X* is encoded into a ⌈*N*/*k*⌉×*D* matrix $\mathcal {X}$ of plaintexts, and the vector of labels *y* is encoded into a vector $\mathcal {Y}$ of plaintexts. These plaintexts are then encrypted and sent to the untrusted party (e.g. cloud service), which performs the homomorphic training computation, resulting in an encrypted logistic regression model. The gradient descent training algorithm over encrypted data (Algorithm 3) is presented below.


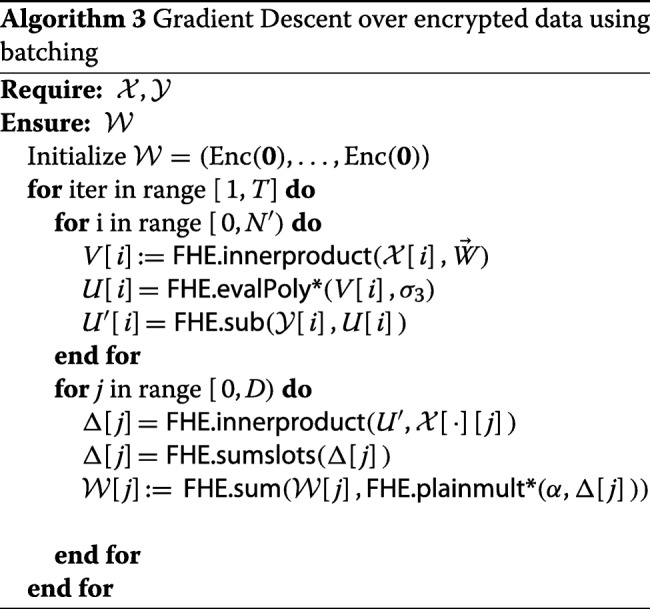


In Algorithm 3, we put a ‘*’ after the evalPoly and plainmult functions to indicate that the corresponding functions are combined with the bootstrapping/scaling function bscale in order to emulate fixed point arithmetic. More details about evaluating *σ*_3_ and multiplying *α* is in ‘incorporating scaling’ section below.

The only other place that requires further explanation is the FHE.sumslots function. The input to this function is a batched encryption of a vector *v*=(*v*_0_,*v*_1_,…,*v*_*k*−1_), and the output is an encryption of $v'= ({\sum \nolimits }_{i} v_{i}, \ldots, {\sum \nolimits }_{i} v_{i})$. In general, this function can be implemented based on the slot rotation functionality. More precisely, our choice of FHE parameters guarantees that we can cyclically rotate the values in an encrypted vector. Note that the number of slots *k* is a divisor of the FHE parameter *n*, hence is always a power of 2. Let *k*=2^*ℓ*^, and let FHE.rotate(c,j) denote the operation of cyclic rotation to the right by *j* slots, i.e., it sends an encryption of (*v*_0_,…,*v*_*k*−1_) to an encryption of (*v*_*k*−1_,*v*_0_,…,*v*_*k*−2_). Then the FHE.sumslots function is as presented in Algorithm 4.


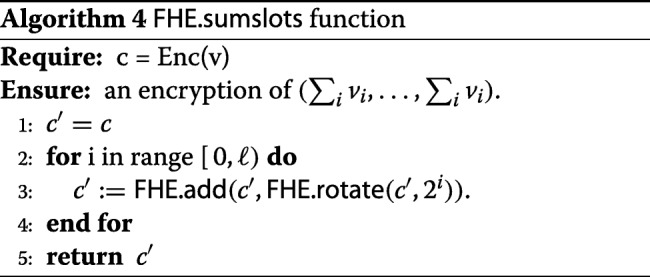


#### **Lemma 1**

Algorithm 4 is correct, i.e. the output *c*^′^ is an encryption of $\Sigma v = ({\sum \nolimits }_{i} v_{i}, \ldots, {\sum \nolimits }_{i} v_{i})$.

#### *Proof*

Since *k*=2^*ℓ*^, we have that the final result *c*^′^ is equivalent to

$$\sum\limits_{i=0}^{k - 1} \textsf{FHE.rotate}(c, i) \,. $$

The claim now follows, since the sum of all rotations of the vector *v* is exactly *Σv*. □

### Optimization techniques

We introduce an optimization to further accelerate our implementation. In the last step of Algorithm 3, the FHE.plainmult operations (see [11]) needs to be performed *D* times. Although these operations themselves are fast, the accompanied homomorphic scaling is expensive. Therefore, we employ an optimization to reduce the number of multiplications from *D* to *D*/*k*. Since *Δ*[*j*] is an encryption of a constant vector, we can combine the content of *k* of those into one ciphertext, encrypting (*δ*_0_,…,*δ*_*k*−1_). Then multiplying this ciphertext by *α* would multiply the values in all slots, resulting in an encryption of (*αδ*_0_,…,*αδ*_*k*−1_). After the multiplication, we can “expand” the result back to *k* ciphertexts, each encrypting a constant vector of *αδ*_*i*_. This expansion step can be implemented via FHE.sumslots. The precise algorithms FHE.combine and FHE.expand are introduced below.


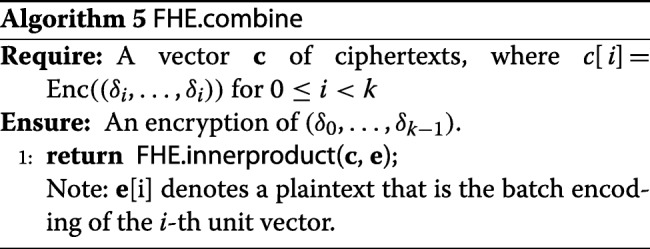



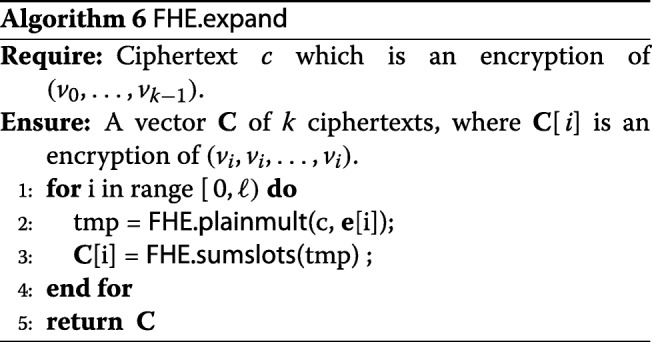


### Incorporating scaling

Some attention to details is needed since the arithmetic system uses fixed point representation.

#### Evaluating *σ*_3_

Recall that *σ*_3_(*x*)=0.5+0.197*x*−0.004*x*^3^, and the representation uses fixed point arithmetic with *p*=127, *l*=1, *f*=1. We will scale 0.5 to ⌊0.5*p*^2^⌋=8064 with a scaling factor of 2. The second coefficient 0.197 will be scaled to ⌊0.197*p*⌋=25. For the coefficient 0.004≈0.063^2^, we scale 0.063 to ⌊0.063*p*⌋=8. Then we can compute *σ*_3_(*x*) over encrypted data in the following way (recall that $\tilde {x} = \lfloor xp \rfloor $):

$$ {}\sigma_{3}(x) \approx \textsf{bscale}(8064 + 25\tilde{x} - \textsf{bscale}(\textsf{bscale}(8 \tilde{x})^{2}) \cdot \tilde{x}). $$

#### Multiplying by learning rate

In the last step of each iteration of the training algorithms, the ciphertext is multiplied by the learning rate *α*. The challenge is that the learning rate we use (*α*=0.002) is so small that it can not be represented by the fixed point representation we use. To see this, note that we have *p*=127 and *f*=1, so the smallest positive number that can be represented is 1/127≈0.008. To resolve this issue, we start by writing $\alpha = (\sqrt {\alpha })^{2}$. Since $\sqrt {0.002} \approx 0.0447$, it can be represented by our fixed point system, as [0.00447*p*]=6. Then we multiply the input by this value twice to obtain the result. After each multiplication, bscale is used to put the underlying number to correct scale. That is:

$$ \alpha x \approx \textsf{bscale}(6 \cdot \textsf{bscale}(6 \cdot \tilde{x})) \,. $$

#### Sign extraction in 1-Bit GD

In order to implement the 1-Bit GD training algorithm, we need a function FHE.signExtract that homomorphically extracts the sign in a fixed point number. Fortunately, this function can be implemented using the bscale function as a subroutine. Since FHE ciphertexts encrypt scaled integers rather than point numbers, it suffices to extract the sign from an signed integer. Moreover, because the sign of an integer is just the most significant digit in its base-*p* expansion, we can extract it directly using bscale(·,*r*−1).

Note that the total degree of this algorithm is *erp*^*e*−*r*+1^, which is smaller than the usual fixed point scaling, which has degree *erp*^*e*−*f*^. This advantage motivates the use of the 1-Bit GD algorithm in our work. The rest of the 1-Bit GD algorithm over encrypted data is exactly the same as Algorithm 3, hence we omit the details.

### Performance results

Table 1 presents the performance results for the iDASH dataset, and Table 2 presents the performance numbers for a subset of the MNIST dataset containing only handwritten digits ‘3’ and ‘8’. In both tables the performance of models trained on plaintext data using MATLAB are compared to models produced by training on encrypted data. We performed the experiments on an Intel(R) Xeon(R) CPU E3-1280 v5 @ 3.70GHz and 16GB RAM. Our experiments use only a single thread, although we note that some of the costliest parts of the computation would be easily parallelizable. We run the same training algorithms on both encrypted and unencrypted data, and compare the results. In order to evaluate the quality of the predictive models obtained, we run a 10-fold cross validation on both training sets, and compute the average Area Under the Curve (AUC) values. Since the unencrypted computation in MATLAB is several orders of magnitude faster than the encrypted computation (less than 1 second), we decided not to compare the unencrypted and encrypted running times side-by-side.

**Table 1:** Running 10-fold cross-validation on the iDASH dataset with 1579 samples and 18 selected genotypes

Training method	# iterations	Avg. training time	Avg. AUC	Avg. AUC (unencrypted)
GD + *σ*_3_	36	115.33 h	0.690	0.690
1-Bit GD + *σ*_1_	36	14.90 h	0.668	0.690

The first average AUC value is obtained from running the training algorithm using SEAL on encrypted data. The second AUC value is obtained from running the same algorithm on unencrypted data using MATLAB

**Table 2:** Running 10-fold cross-validation on compressed MNIST dataset with 1500 samples and 196 features

Training method	# iterations	Avg. training time	Avg. AUC	Avg. AUC (unencrypted)
GD + *σ*_3_	10	48.76 h	0.974	0.977
1-Bit GD + *σ*_1_	10	27.10 h	0.974	0.978

The first average AUC value is obtained from running the training algorithm using SEAL on encrypted data. The second AUC value is obtained from running the same algorithm on unencrypted data using MATLAB

The algorithms, when operated on encrypted data, were able to obtain almost identical accuracy compared to training on unencrypted data. Obviously training on encrypted data is much slower than training on unencrypted data, which can be acceptable in some use-cases, and unacceptable in others; for the datasets that we used, training can take between half a day to few days, although substantial improvements in computational performance can be expected by improving our implementation, and extending it to use multiple threads.

## Discussion

In this work we presented new ways to train Logistic Regression over encrypted data, which allow an arbitrary number of iterations due to FHE bootstrapping, thus making our models updatable once new data becomes available without requiring decryption at any point; this is different from other recently proposed approaches that limit the number of iterations in the training process. The time per iteration scales linearly with the data size. Hence, the total time for training *N* samples with *D* features per sample using *T* iterative steps over encrypted data is a linear function in the product *N*·*D*·*T*. Therefore, our solutions scale gracefully with the size of the data. Moreover, many of the ideas presented here can be used for training other machine learning models, for example Neural Networks, by using polynomial approximations to the activation functions.

## Conclusions

There is a growing interest in applying machine learning algorithm to private data, such as medical data, genomic data, financial data, and more. For critical applications homomorphic encryption can guarantee the highest level of data privacy during computation, but it also comes with a high cost, especially in terms of computation time.
